# Digitalisierung im Gesundheitswesen und ihre Effekte auf die Qualität der Gesundheitsversorgung

**DOI:** 10.1007/s00103-022-03493-3

**Published:** 2022-02-18

**Authors:** Jan Benedikt Brönneke, Jörg Felix Debatin

**Affiliations:** health innovation hub (hih), Bundesministerium für Gesundheit, Torstr. 223, 10115 Berlin, Deutschland

**Keywords:** Digitale Tools, Gesundheitsanwendungen, Qualitätssicherung, Versorgungsqualität, Digital tools, Health applications, Quality assurance, Quality of care

## Abstract

Die Sicherung der Qualität von Gesundheitsleistungen ist ein Kernanliegen des Systems der gesetzlichen Krankenversicherung. Es ist daher nicht überraschend, dass die jüngeren Initiativen zur (weiteren) Digitalisierung des Gesundheitssystems ohne Ausnahme mit der Sicherung oder gar Steigerung der Qualität der Leistungserbringung begründet werden. Dies betrifft beispielsweise die Einführung der elektronischen Patientenakte und anderer Anwendungen der Telematikinfrastruktur mit dem Patientendaten-Schutz-Gesetz (PDSG), aber auch die Einführung der digitalen Gesundheitsanwendungen mit dem Digitale-Versorgung-Gesetz (DVG) sowie die Stärkung der Krankenhaus-IT mit dem Krankenhauszukunftsgesetz (KHZG).

Der Artikel beleuchtet die Frage, in welchem Verhältnis der Einsatz verschiedener digitaler Lösungen zu den klassischen Zielen der Qualitätssicherung in der Gesundheitsversorgung steht, insbesondere ob digitale Lösungen geeignet sind, Qualitätssicherung zu befördern.

Es zeigt sich, dass digitale Lösungen grundsätzlich geeignet sind, Qualität zu sichern. Dies ist auf 2 Charakteristika digitaler Lösungen zurückzuführen: die Möglichkeit zur umfassenden Erhebung von Daten und die Möglichkeit der zeit- und ortsunabhängigen Bereitstellung von Daten. Es zeigt sich aber auch, dass der Nutzen digitaler Lösungen vom konkreten Anwendungsfall abhängig ist.

## Abstract (en)

Ensuring the quality of healthcare services is a core concern of the statutory health insurance system. It is therefore not surprising that recent initiatives to (further) digitalize the healthcare system, specifically to introduce the electronic patient record and other applications in the telematics infrastructure, with the Patient Data Protection Act (“Patientendaten-Schutz-Gesetz”, PDSG) of digital health applications with the Digital Care Act (“Digitale-Versorgung-Gesetz”, DVG), or to strengthen hospital IT with the Hospital Future Act (“Krankenhauszukunftsgesetz”, KHZG), are justified by ensuring or even increasing the quality-of-service provision.

This article sheds light on the question of how the use of various digital solutions relates to the classic goals of quality assurance in healthcare, in particular, whether digital solutions are suitable for promoting quality assurance.

It is shown that digital solutions are fundamentally suitable for ensuring quality. This is due to two characteristics of digital solutions: the possibility of comprehensive data collection and the possibility of making data available independent of time and place. However, it is also apparent that the benefits associated with digital solutions are highly dependent on the specific use cases.

## Einleitung

Qualität hat im deutschen Gesundheitswesen einen hohen Stellenwert. So nennt das Fünfte Buch Sozialgesetzbuch (SGB V) Begriffe wie „Qualität“, „Qualitätssicherung“, „Qualitätsmanagement“ fast 400-mal in verschiedenen Kontexten:

- Leistungen der gesetzlichen Krankenversicherung (GKV) haben in ihrer Qualität dem allgemein anerkannten Stand der medizinischen Erkenntnisse zu entsprechen (§ 2 Absatz 1 Satz 3 SGB V),
- Wettbewerb zwischen Krankenkassen soll der Verbesserung der Qualität von Leistungen dienen (§ 4a Absatz 1 Satz 1 SGB V),
- die Kassenärztliche Bundesvereinigung und der Spitzenverband Bund der Krankenkassen können im Bundesmantelvertrag Qualitätszuschläge vereinbaren (§ 87 Absatz 2b Satz 4),
- der Gemeinsame Bundesausschuss (G-BA) beschließt verbindliche Richtlinien zur Qualitätssicherung (§ 92 Absatz 1 Satz 2 Nummer 13), inklusive Richtlinien zur Qualitätsbeurteilung in der vertragsärztlichen Versorgung (§ 135b Absatz 2 Satz 2 SGB V), zu Qualitätsberichten im Krankenhaus, Mindestmengen und anderen Qualitätssicherungsmechanismen im Krankenhaus (§ 136b SGB V).

Mit dem Gesetz wurde außerdem die Gründung des Instituts für Qualität und Wirtschaftlichkeit im Gesundheitswesen (IQWiG, nach § 139a SGB V) und des Instituts für Qualitätssicherung und Transparenz im Gesundheitswesen (IQTIG, nach § 137a SGB V) vorgeschrieben. Diese Institute nehmen inzwischen verschiedene Aufgaben zur Messung, Darstellung und Bewertung von qualitätsrelevanten Parametern wahr – mit zum Teil erheblichen Implikationen für die jeweils adressierten Marktteilnehmer. Ganz grundsätzlich sind überdies die Leistungserbringer, also Vertragsärzte, Krankenhäuser, medizinische Versorgungszentren etc. verpflichtet, ein internes Qualitätsmanagement (QM) einzuführen und sich an einrichtungsübergreifenden Maßnahmen der Qualitätssicherung (QS) zu beteiligen, mit dem Ziel der Verbesserung der Ergebnisqualität (§ 135a Absatz 2 SGB V).

Abseits rechtlicher Verpflichtungen kann auch ein intrinsisches Interesse vieler Institutionen im Gesundheitswesen bestehen, Qualitätsmanagement zu betreiben. Letztlich sind Qualitätssicherung und -management Methoden, die in anderen Wirtschaftszweigen zur Steigerung der Effizienz entwickelt und eingesetzt werden, also die Wettbewerbsfähigkeit steigern [1]. Darüber hinaus kann dadurch das Risiko möglicher zivilrechtlicher Schadensersatzansprüche (von Patienten) verringert werden. Insofern verwundert es nicht, dass sich neben gesetzlich vorgesehenen Qualitätssicherungsmechanismen auch private Initiativen diesem Ziel verschrieben haben (wie etwa die Initiative Qualitätsmedizin).

Eine qualitativ hochwertige Versorgung der Versicherten kann mithin als eine der grundsätzlichen Zielgrößen des Gesundheitswesens angesehen werden. In Anbetracht dessen und vor dem Hintergrund der jüngsten gesetzgeberischen Maßnahmen zur Beförderung der Digitalisierung des Gesundheitssystems (z. B. dem Terminservice- und Versorgungsgesetz – TSVG, dem Digitale-Versorgung-Gesetz – DVG, dem Krankenhauszukunftsgesetz – KHZG, dem Patientendaten-Schutz-Gesetz – PDSG und dem Digitale-Versorgung-und-Pflege-Modernisierungs-Gesetz – DVPMG) stellt sich die Frage, ob die Digitalisierung im Gesundheitswesen auch der Steigerung der Qualität der Gesundheitsversorgung dient. So zumindest wird es vielfach postuliert [2–4], zum Teil aber auch in Abrede gestellt [5, 6]. In der öffentlichen Diskussion wird dabei der Begriff der Qualitätssteigerung bisweilen sehr allgemein gefasst, ohne jedoch konkrete Konzepte des Qualitätsbegriffs im Gesundheitswesen zugrunde zu legen [7, 8].

Der Aufsatz stellt Konzepte der Qualitätssicherung im Gesundheitswesen dar und zeigt anhand von Beispielen, wie der Einsatz digitaler Technologien in den Kontext dieser Konzepte gestellt werden kann.^1^ Ausgehend von diesen Beispielen soll die Frage beantwortet werden, ob die Annahme der positiven Effekte von digitalen Lösungen auf die Qualität grundsätzlich haltbar ist. Schließlich soll induktiv diskutiert werden, welche Eigenart digitaler Technologien diese für Qualitätssicherung besonders geeignet erscheinen lässt.

## Qualität, Qualitätsmanagement und Qualitätssicherung in der Gesundheitsversorgung

Trotz der vielfachen Forderung des Gesetzgebers, Qualität sicherzustellen, findet sich im SGB V keine Legaldefinition der Begriffe Qualität, Qualitätssicherung, Qualitätsmanagement etc. [9]. Relevant sind daher grundlegende Konzepte zur Qualität in der Medizin. Die heute – in verschiedenen Adaptionen [10, 11] – gebräuchliche Definition der Qualität ist diejenige der Norm DIN EN ISO 9000:2005, in der Qualität definiert ist, als der „Grad, in dem ein Satz inhärenter Merkmale Anforderungen erfüllt“. Die wohl älteste bis heute gebräuchliche Definition der Qualität im Gesundheitswesen stammt von Donabedian und ist der genannten Definition der ISO-Norm sehr ähnlich: „Quality of care is the extent to which actual care is in conformity with preset criteria for good care“ [12]. Diesen Definitionen der Qualität sind 3 wesentliche Bestandteile gemein:

1. ein festgelegter Sollzustand („Anforderungen“, „preset criteria“),
2. ein zu bemessender Istzustand („Satz inhärenter Merkmale“, „actual care“) und
3. eine graduelle Bemessung der Relation von Ist- zu Sollzustand („Grad [der Erfüllung]“, „extent [of conformity]“).

Für die Definitionen von Qualitätsmanagement und Qualitätssicherung kann ebenfalls auf die DIN EN ISO 9000:2005 zurückgegriffen werden: Qualitätsmanagement ist dort definiert, als „aufeinander abgestimmte Tätigkeiten zum Leiten und Lenken einer Organisation bezüglich Qualität“. Qualitätssicherung ist als derjenige Teil des Qualitätsmanagements definiert, der auf das „Erzeugen von Vertrauen darauf gerichtet ist, dass Qualitätsanforderungen erfüllt werden“. Beide Maßnahmen bedürfen der Qualitätsmessung und -bewertung auf der Basis von objektivierbaren Qualitätsindikatoren [13].

Üblicherweise wird hier nach Donabedian darüber hinaus zwischen Strukturqualität, Prozessqualität und Ergebnisqualität unterschieden [14], eine Unterscheidung die auch das SGB V implizit, zum Teil auch explizit vornimmt, etwa in § 136 Absatz 1 Satz 1 Nummer 2. *Strukturqualität* bezieht sich auf Verfügbarkeit und Beschaffenheit der Strukturen, innerhalb derer Leistungen erbracht werden. Dies umfasst z. B. Qualifikation und Anzahl des verfügbaren (ärztlichen) Personals, vorhandene (digitale) Infrastrukturen, finanzielle und andere Ressourcen, Verfügbarkeit relevanter Informationen in medizinischen Datenbanken etc. *Prozessqualität* bezieht sich auf die konkreten Prozesse, die konkrete Art und Weise der Leistungserbringung, also z. B. das Einhalten von etablierten oder vorgeschriebenen prozessualen Standards wie ärztlichen Leitlinien, die Nutzung von konkreten Ablaufplänen, Checklisten etc. Die *Ergebnisqualität* schließlich bezieht sich auf das konkrete (Be)Handlungsergebnis, also insbesondere die objektive messbare Verbesserung des Gesundheitszustandes oder auch die subjektive Zufriedenheit der Patienten [15]. Dieser Dreiteilung liegt die Vermutung eines Wirkzusammenhangs zugrunde: Qualitativ hochwertige Strukturen schaffen die Voraussetzungen für qualitativ hochwertige Prozesse, diese wiederum sind Voraussetzung für ein qualitativ hochwertiges Ergebnis – wobei das Letztgenannte das eigentliche Ziel darstellt [16]. Die Messung und Bewertung gerade der Ergebnisqualität gestalten sich dabei besonders schwierig [9], weswegen Struktur- und Prozessqualität vielfach als Surrogatparameter genutzt werden [13].

Vor dem Hintergrund der Notwendigkeit von Qualitätsmessung und -bewertung liegt jedenfalls nahe, dass digitale Lösungen, in deren Kern ja gerade die ständige Verarbeitung von Daten liegt, hierbei hilfreich sein können und entsprechend positiv auf die Qualität der Gesundheitsversorgung einwirken.

## Digitale Lösungen und Strukturqualität

Digitale Lösungen werden bereits seit Langem zur Verbesserung der Strukturqualität von Versorgungsleistungen eingesetzt. Zuvorderst ist hier an den Einsatz von Software in der Verarbeitung von versorgungsrelevanten Daten zu denken, die digitale Prozesse ermöglicht. Dazu gehören digitales Patientenstammdatenmanagement ebenso wie Kommunikation zwischen Leistungserbringern via (verschlüsselter) E‑Mail, Einsatz von QM-Software, digitale Dokumentation von Medikation, Nutzung von Schnittstellen zwischen Praxisverwaltungssystemen und den Systemen von medizinischen Laboren zum Austausch von Labordaten [17]. Ebenfalls inkludiert sind die Infrastruktur zur Erbringung telemedizinischer Leistungen (die gerade in der COVID-19-Pandemie von großer Bedeutung sind [18]), der Zugang zu digitalen Datenbanken zur Einsicht in aktuelle klinische Studien und digitale Berichterstattungssysteme zur anonymen Meldung von kritischen Ereignissen* (Critical Incident Reporting – CIRS; *[19]). Insbesondere elektronische Patientenakten und andere Systeme zum Austausch von Gesundheitsdaten [20] sind bereits heute in Krankenhäusern und Arztpraxen – in verschiedener Ausprägung – zu finden. Die mit dem Einsatz solcher Lösungen verbundenen direkten Netzwerkeffekte schaffen die Grundlage für einen schnelleren und sichereren Informationsaustausch zwischen involvierten Leistungserbringern und damit für effizientere Leistungsprozesse [21]. Letztere tragen wiederum zu einer Verbesserung von Versorgungsqualität bei [22–24].

Freilich sind diese Effekte noch überwiegend auf relative „Inseln“ beschränkt, etwa auf die Kommunikation zwischen veranlassenden Ärzten und medizinischen Laboren oder innerhalb eines Krankenhauses. Überdies werden digitale Infrastrukturen noch überwiegend für die Übermittlung und Verarbeitung von administrativen Daten genutzt. Die derzeitige Herausforderung besteht in der Schaffung einer leistungs- und leistungserbringerübergreifenden Infrastruktur, insbesondere auch zur longitudinalen Verarbeitung behandlungsrelevanter medizinischer Daten. Die Schaffung von Infrastrukturen zur Verarbeitung solcher Daten könnte zum einen über die zeitnahe Bereitstellung behandlungsrelevanter Daten, zum anderen aber auch über die Bereitstellung für die Forschung der Verbesserung der Qualität der Gesundheitsversorgung dienen. Diese Ziele werden unter anderem auch mit der Schaffung der Telematikinfrastruktur und der elektronischen Patientenakte verfolgt [25].

## Digitale Lösungen und Prozessqualität

Bezüglich der Prozessqualität von Gesundheitsleistungen kommt der Einsatz digitaler Technologien insbesondere bei der Sicherung der Einhaltung von Standards zum Tragen. Es handelt sich in erster Linie um medizinische Leitlinien und andere Regelwerke, für die in meist evidenzbasierten klinischen Studien nachgewiesen wurde, dass ihre Einhaltung mit besseren klinischen Ergebnissen korreliert oder sogar kausal zusammenhängt [26]. Neben digitalen Checklisten, die statt papierbasierten Listen genutzt werden [27, 28], bestehen verschiedene Lösungen, wie z. B. die grundsätzliche Nutzung von Dashboards (grafische Benutzeroberflächen zur Datenvisualisierung) zur Steigerung der Einhaltung von Leitlinien [29] und auch für spezifische Anwendungsfälle, wie etwa in Notaufnahmen [30]. Darüber hinaus ist auch der Einsatz von indikations- und prozedurenspezifischen digitalen QS-Tools möglich, insbesondere dort, wo ohnehin mit digital gesteuerten Medizingeräten gearbeitet [31] oder anderweitig Prozesse bereits durch den Einsatz von Software geprägt sind, wie etwa in der Pathologie [32, 33].

Auch hier besteht die Herausforderung, Prozessqualität leistungserbringerübergreifend zu sichern. Auch wenn mit strukturierten Behandlungsprogrammen (§ 137f SGB V, sogenannte Disease-Management-Programme, DMP) qualitätsfokussierte, „analoge“ Versorgungsansätze bestehen [34], ist die Nutzung digitaler Lösungen in diesen Fällen bislang nicht explizit vorgesehen. So hat der G‑BA zum Beispiel bislang keinen Beschluss zur Aufnahme „geeigneter digitaler medizinischer Anwendungen“ (§ 137f Absatz 8 SGB V) gefasst, obwohl bereits erste digitale Gesundheitsanwendungen (DiGA) im DiGA-Verzeichnis für DMP-Indikationen (vorläufig) aufgenommen wurden. Von Interesse ist, dass mit § 8 Absatz 3 der Digitale-Gesundheitsanwendungen-Verordnung (DiGAV) die Koordination der Behandlungsabläufe sowie die Ausrichtung der Behandlung an Leitlinien und anerkannten Standards, also 2 klassische Belange der Sicherung von Prozessqualität, explizit als positive Versorgungseffekte von DiGA anerkannt sind. Hier hat der Verordnungsgeber also einen neuen Weg mittelbarer, nämlich über die Patienten laufender [35], digitaler QS von Prozessen der Leistungserbringer ermöglicht.

## Digitale Lösungen und Ergebnisqualität

Als der herausforderndste Teil der Qualitätssicherung in der Gesundheitsversorgung stellt sich die Sicherung der Ergebnisqualität dar. Überwiegend ist die Sicherung der Ergebnisqualität mit verschiedenen Bewertungsverfahren als *Ex-ante*-Prozess gestaltet, findet also vor der eigentlichen Anwendung statt: insbesondere die Bewertung für neue diagnostische und therapeutische Methoden nach § 135 SGB V, die Bewertung des Nutzens von Arzneimitteln mit neuen Wirkstoffen nach § 35a SGB V, aber auch die Arzneimittelzulassung und Konformitätsbewertung von Medizinprodukten nach europäischen Vorschriften.

Bevor Methoden oder Produkte in der Gesundheitsversorgung angewandt werden dürfen oder von der GKV vergütet werden, ist im Rahmen dieser Verfahren der (medizinische) Nutzen der Methoden und Produkte nachzuweisen. Dies geschieht den Grundsätzen der evidenzbasierten Medizin folgend überwiegend mittels randomisierter klinischer Studien (Randomized Controlled Trials – RCT). Diese sind aufgrund ihres experimentellen Charakters und des klar definierten Studiensettings, innerhalb dessen sich ein *Ceteris-paribus*-Zustand, also ein Zustand unter gleichen Bedingungen, am ehesten erreichen lässt, mit ihrer hohen internen Validität am besten geeignet, potenziell kausale Zusammenhänge zwischen Anwendung der Methode oder des Produkts und der Verbesserung patientenrelevanter Endpunkte (Mortalität, Morbidität, gesundheitsbezogene Lebensqualität und Nebenwirkungen) nachzuweisen [36]. Dessen ungeachtet sind in jüngerer Zeit – nicht zuletzt aufgrund der Möglichkeiten der Digitalisierung – zunehmend auch Studientypen in den Mittelpunkt gerückt, die auf versorgungsnahen Echtweltdaten basieren (Real World Evidence – RWE, basierend auf Real World Data – RWD). Obwohl solche RWE für gewöhnlich über eine geringere interne Validität als RCTs verfügt, hat sie eine hohe externe Validität und ermöglicht damit eher Rückschlüsse über den Nutzen für konkrete Patienten in konkreten Lebens- und Behandlungssituationen [37]. Hier können digitale Lösungen einen zentralen Beitrag zur Erfassung von patientenrelevanten Outcomes leisten. Zu denken ist dabei an die Nutzung von „smart wearables“ (Armbänder zur Erfassung von Vitalfunktionen), die beispielsweise im Monitoring [38], aber auch im Rahmen von klinischen Studien [39] durch beziehungsweise am Patienten eingesetzt werden, oder (Smartphone‑)Applikationen zur Erfassung von patientengenerierten Daten zur Messung von Outcomes (Patient-reported Outcomes Measures – PROM; [40]).

Auch DiGA sind bezüglich der Ergebnisqualität in mehrfacher Hinsicht von Interesse: Zum einen umfasst die Definition der DiGA in § 33a SGB V auch die Anwendung „in der Versorgung durch Leistungserbringer“, was einen unmittelbaren uni- oder bidirektionalen Informationsfluss zwischen Leistungserbringer und Patient ermöglicht, der die Abfrage patientenrelevanter Outcomes beinhalten kann. Zum anderen – und dies ist ein Novum im ansonsten auf einmalige Bewertung ausgerichteten Gesundheitssystem – ist den Vertragspartnern nach § 134 Absatz 1 SGB V, also GKV-Spitzenverband und dem jeweiligen DiGA-Hersteller vom Gesetzgeber explizit aufgegeben, dass die Vereinbarungen über Vergütungsbeträge auch erfolgsabhängige Preisbestandteile umfassen sollen. Damit ist der DiGA die kontinuierliche QS gleichsam in die Wiege gelegt. Hier bleibt abzuwarten, wie die Vorgabe, von der auch abgewichen werden kann („Sollvorschrift“), in der Praxis umgesetzt wird.

## Fazit

Der Einsatz digitaler Technologien kann grundsätzlich positive Effekte auf die Qualität der Gesundheitsversorgung haben. Diese Effekte lassen sich auch in der Logik der konkreten, bestehenden Qualitätskonzepte für Struktur‑, Prozess- und Ergebnisqualität abbilden und nachweisen. Das Postulat der Qualitätssteigerung durch den Einsatz digitaler Technologien ist damit jedenfalls grundsätzlich haltbar. Abseits der hier aufgeführten exemplarischen Anwendungsfälle lässt sich aber auch zeigen, dass digitale Lösungen nicht bei allen Anwendungsfällen zwingend zur Qualitätssicherung führen (z. B. in Kontexten, in denen zu häufig zeitkritische und von Standardprozeduren abweichende Entscheidungen getroffen werden müssen [41]). Auch ist die vorhandene Evidenz für positive Effekte von digitalen Lösungen auf die Qualität der Gesundheitsversorgung derzeit häufig noch wenig belastbar [42–44]. Die pauschale Annahme einer Qualitätssteigerung und -sicherung durch den Einsatz digitaler Technologien in allen Einsatzszenarien ist mithin nicht gerechtfertigt. Vor diesem Hintergrund wäre dem Diskurs eine differenzierte, sich an konkreten Qualitätskonzepten orientierende Betrachtung sicherlich zuträglich. Wünschenswert wäre eine systematische Analyse der Qualitätsrelevanz digitaler Lösungen im Gesundheitssystem um die Nutzung von „nützlichen“ Lösungen zu fördern (wie es etwa mit dem KHZG für den stationären Bereich versucht wird [45]).

Schließlich stellt sich die Frage, ob aus den genannten Beispielen abgeleitet werden kann, in welchem Kontext sich digitale Lösungen zur Qualitätssteigerung eignen. Den exemplarischen Anwendungsfällen digitaler Lösungen in der Gesundheitsversorgung sind die Erfassung und Bereitstellung von Daten gemein. Sowohl in Hinblick auf Struktur- als auch Prozessqualität konnte für verschiedene Anwendungsfälle gezeigt werden, dass mittels digitaler Lösungen Daten zur richtigen Zeit am richtigen Ort zur Verfügung gestellt werden können. Dies betrifft sowohl Strukturen zum Austausch patientenbezogener Informationen, die im konkreten Behandlungskontext gebraucht werden (zum Beispiel Labordatenaustausch oder elektronische Patientenakten), als auch prozessbezogene Informationen zum medizinisch „richtigen“, nämlich evidenzbasierten Vorgehen (zum Beispiel digitale Checklisten oder Dashboards). Auch zur Sicherung (und Steigerung) der Ergebnisqualität können digitale Lösungen beitragen: Hier geht es primär um die longitudinale Erfassung von patientenrelevanten Daten (zum Beispiel über „smart wearables“), die bislang kaum oder nur punktuell erfasst werden können. In dieser umfassenden Erhebung von Daten und der Möglichkeit der zeit- und ortsunabhängigen Bereitstellung von Daten scheint der eigentliche Wert digitaler Lösungen für die Qualität der Gesundheitsversorgung zu liegen (so wohl auch [46]). Dies erscheint vor dem Hintergrund der für die Sicherung und Steigerung von Qualität absolut erforderlichen Qualitätsmessung und -bewertung auch plausibel. Auch erklärt dies, warum sich der Einsatz digitaler Technologien in neuartigen Situationen, in denen bestehende Daten keinen Mehrwert liefern, nicht qualitätssteigernd auswirkt. Sollte sich dieser Eindruck durch weitere Studien belegen lassen, wäre dies ein relevanter Aspekt bei der Erarbeitung weiterer (gesetzgeberischer oder privater) Digitalisierungsmaßnahmen. Letztlich ist der Einsatz digitaler Technologien kein Selbstzweck, sondern muss stets der besseren Versorgung von Patienten und Versicherten dienen.

## Footnotes

1 Über die im Folgenden aufgeführten, rein auf Qualität fokussierten Konzepte hinaus, bestehen auch gesundheitsökonomische Konzepte, die Qualität im Verhältnis zu den entstandenen Kosten zu erfassen suchen, insbesondere um Aussagen über die Kosteneffizienz treffen zu können. Auch wenn die Fragen der Kosteneffizienz von digitalen Lösungen häufig aufgeworfen werden (siehe etwa [47]), bedürften diese einer eigenen Betrachtung und werden im vorliegenden Artikel nicht berücksichtigt.
